# When Tuberculosis Comes Back: Who Develops Recurrent Tuberculosis in California?

**DOI:** 10.1371/journal.pone.0026541

**Published:** 2011-11-01

**Authors:** Lisa Pascopella, Kathryn DeRiemer, James P. Watt, Jennifer M. Flood

**Affiliations:** 1 Tuberculosis Control Branch, Division of Communicable Disease Control, Center for Infectious Diseases, California Department of Public Health, Richmond, California, United States of America; 2 School of Medicine, University of California Davis, Davis, California, United States of America; 3 Division of Communicable Disease Control, Center for Infectious Diseases, California Department of Public Health, Richmond, California, United States of America; McGill University, Canada

## Abstract

**Background:**

Recurrent tuberculosis suggests potentially modifiable gaps in tuberculosis treatment and control activities. The frequency of late recurrences following treatment completion has not been well-studied. We determined the frequency of, and risk factors associated with, tuberculosis that recurs at least one year after completion of anti-tuberculosis therapy in California.

**Methods:**

The study population included culture-positive, pulmonary tuberculosis patients reported to the California tuberculosis case registry from 1993 to 2007 who completed anti-tuberculosis therapy. A person with late recurrent tuberculosis was defined as an individual that appeared in the registry more than once, determined by match on name and date-of-birth, with at least one year between treatment completion of the first episode and treatment initiation of the second episode.

**Results:**

Among 23,517 tuberculosis patients, 148 (0.63%) had a late recurrence. Independent risk factors for recurrence included: infection with a pyrazinamide mono-resistant isolate (adjusted hazard ratio, 2.93; p = 0.019); initiation of an isoniazid- and rifampin-only treatment regimen (adjusted hazard ratio, 2.55; p = 0.0412); sputum smear-positive disease (adjusted hazard ratio, 1.96; p = 0.0003); human immunodeficiency virus infection (adjusted hazard ratio, 1.81; p = 0.0149); and birth in the United States (adjusted hazard ratio, 1.88; p = 0.0002). Infection with an isoniazid mono-resistant isolate was protective (adjusted hazard ratio, 0.25; p = 0.0171).

**Conclusions:**

The low frequency of late recurrent tuberculosis in California suggests that local TB control programs are largely successful at preventing this adverse outcome. Nonetheless, we identified subpopulations at increased risk of late tuberculosis recurrence that may benefit from additional medical or public health interventions.

## Introduction

Recurrent tuberculosis (TB) within a population suggests potentially modifiable gaps in TB treatment and control activities. We examined TB recurrence in California to assess its frequency and to identify populations at increased risk that may benefit from specific interventions. A recurrence of TB can be due to relapse or re-infection [1]. To prevent relapse, TB treatment guidelines in the United States (U.S.) recommend extended treatment for TB cases with cavities on chest radiograph and delayed bacterial clearance from sputum [2]. Re-infection is prevented when TB transmission is averted. Populations at high risk of recurrent TB, regardless of the recurrence mechanism, provide an important group for early TB case-finding in high incidence settings [3]. In low incidence settings, targeted case-finding among populations at high risk of recurrence could facilitate TB elimination by preventing transmission. However, the value of additional follow-up of TB patients depends on the probability of recurrence.

Although clinical trials have demonstrated the rarity of TB relapse and re-infection in the U.S. and Canada [4], [5], these studies may not accurately reflect the incidence of recurrent TB under usual TB program conditions. We conducted a large population-based study of recurrent TB to provide a more robust estimate of its frequency and associated risk factors. Because recurrent cases within one year of treatment completion are defined as relapses and are not reported as separate episodes in the U.S., we evaluated persons who had a second TB episode after at least one year following treatment completion of their previous episode. Thus, “late” recurrent TB, as defined in this study, did not include second episodes identified within twelve months after treatment completion, which is the most common period monitored in clinical treatment trials [6]. Late recurrences, not previously measured in large population-based studies, are important because TB patients are not typically followed beyond one year past treatment. If patient subgroups are at increased risk of developing TB, enhanced follow-up of these patients could prevent TB transmission.

## Methods

The study population included culture-positive, pulmonary TB cases reported in California from January 1, 1993 to December 31, 2007. Cases were excluded if they did not complete treatment or if initial drug susceptibility test results were not recorded. Persons with late recurrent TB were those who had more than one episode of culture-positive, pulmonary TB with at least one year between the date treatment was completed for the first TB episode and the date that treatment was initiated for the second TB episode. We identified late recurrent TB by a deterministic match using first name, last name, and date of birth. Minor mismatches were tolerated in names or date of birth if sex, race, ethnicity, and country of origin matched.

The local and state TB case registries were matched to the local and state Acquired Immunodeficiency Syndrome (AIDS) registries to determine HIV status of TB cases. To identify genotypes of pairs of *M. tuberculosis* isolates from patients with late recurrent TB, the national TB genotyping surveillance database, initiated in 2004 [7], was queried. Drug resistance patterns of isolates from first and second TB episodes were investigated from each TB case report.

We assessed changes in the frequency of late recurrences over time with the Joinpoint Regression Program [8]. Statistical analyses were performed using SAS version 9.1 (SAS Institute Inc. Cary, North Carolina, USA). The main outcome variable was a late recurrence, and time-to-recurrence was modeled using Cox regression. Among sets of two dichotomous covariates that were correlated (phi coefficient >0.3), only one variable of the set was chosen as a predictor variable in the multivariate model. Decisions to include one covariate among two or more correlated covariates were based on efficiency (e.g. birth in U.S. was correlated with more than one covariate) and the potential to subsequently implement public health interventions among persons having the characteristic measured by the covariate.

**Figure 1:**
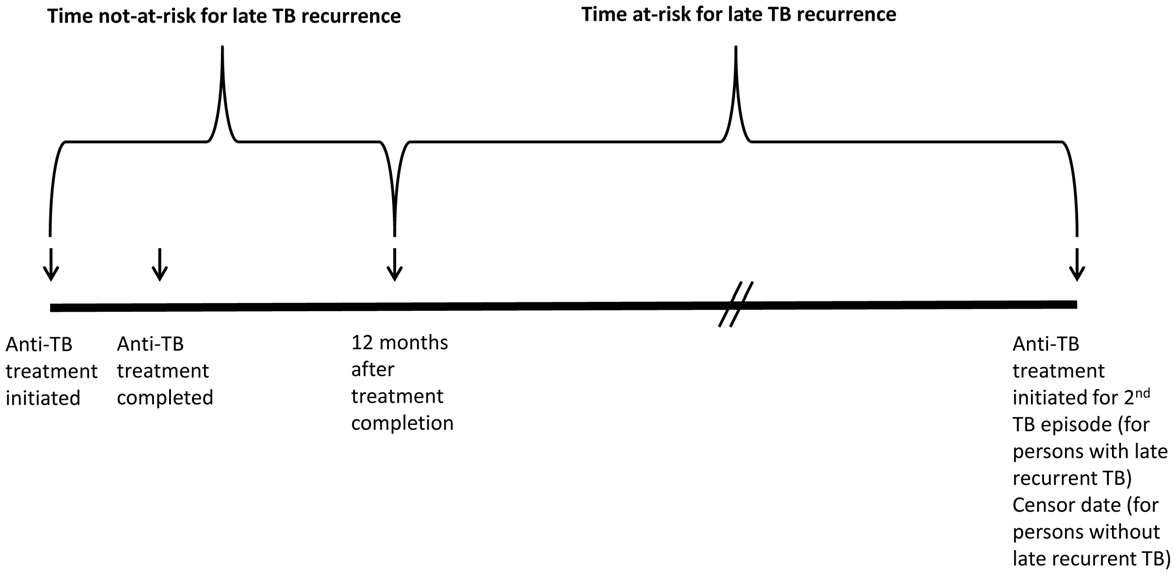
Time-at-risk for late recurrent TB. The diagram depicts times related to treatment initiation and completion that define time-at-risk and time-not-at-risk for late recurrent TB.

A multivariate Cox model was constructed by forwards stepwise selection, and included, a priori, the variable for age at TB report and covariates known to be associated with TB relapse: HIV co-infection, and sputum smear-positive disease. Additional covariates were added if, in univariate analysis, p≤0.2 for association with late recurrence. Age at TB report was retained in the multivariate model. All other covariates were retained in the multivariate model if their p-values were less than 0.05. We tested interaction terms to determine whether they significantly improved the models. We used a log-rank test of the likelihood ratio to determine best model fit. Person-time-at-risk for late recurrence was the time between the date therapy was completed for the first (or single) episode, and the date that therapy started for the second episode or the censor date, December 31, 2006 (to allow one year for a recurrence through December 31, 2007) (Figure 1).

We tested the proportional hazards assumption by comparing cumulative hazards of the multivariate model for strata of each dichotomous variable and by Schoenfeld residual analysis for age. To illustrate the increased risk of late recurrent TB for various subpopulations, we modeled the proportion and timing of recurrences for a 30-year-old population with a progressive increase in numbers of risk factors.

## Results

### Frequency and description of recurrences

**Figure 2:**
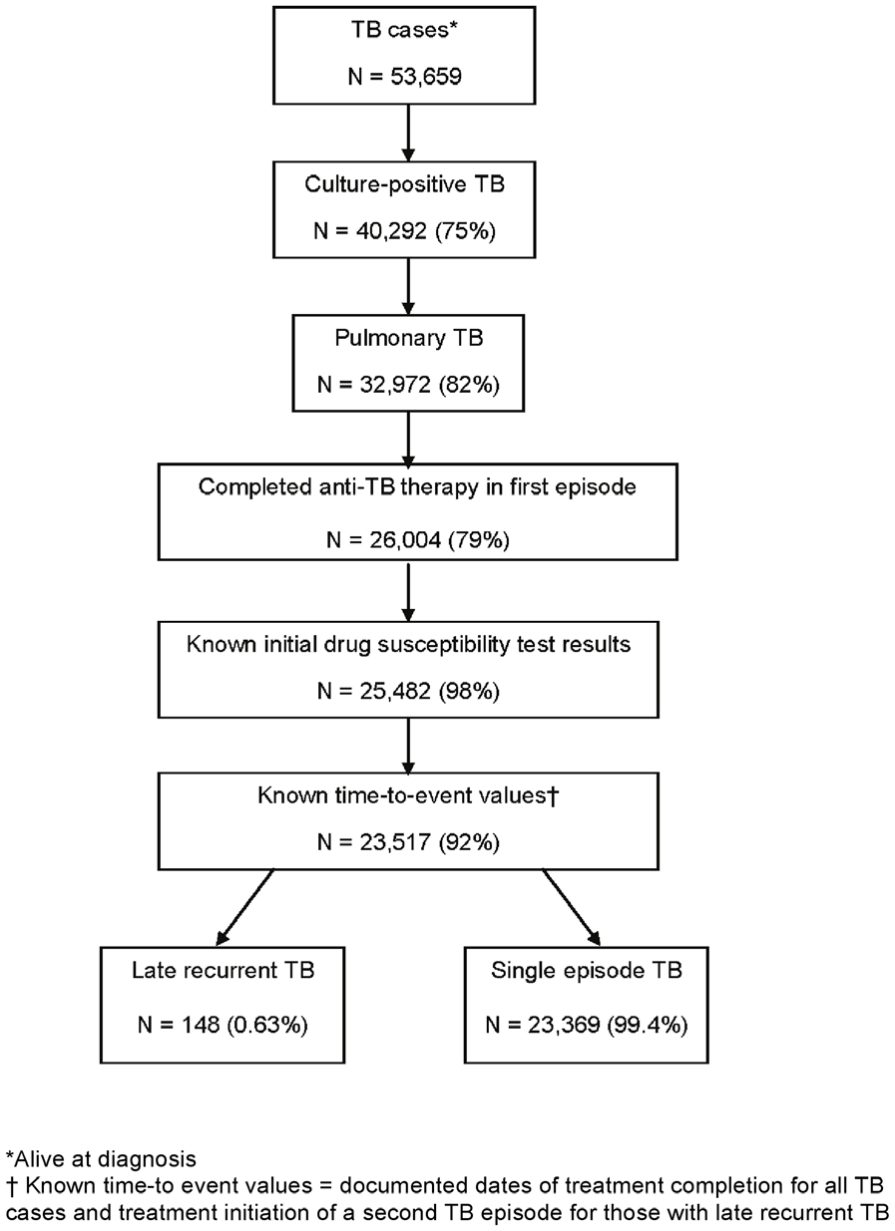
Study population, 1993–2007. The figure shows the classification of patients in the study and the inclusion criteria. TB patients included those who were alive at TB diagnosis with culture-positive, pulmonary TB (in both episodes for recurrent TB patients), and, had completed anti-TB therapy. Patients were excluded if initial isoniazid or rifampin drug susceptibility test results were not documented, or, if the date of treatment completion or date of initiation of treatment for a second TB episode was not documented. Percentages were calculated using the previous group (previous box) as the denominator. One hundred forty eight persons in this study population had late recurrent TB, and 23,369 had one episode of TB (see text for details). TB = tuberculosis.

**Figure 3:**
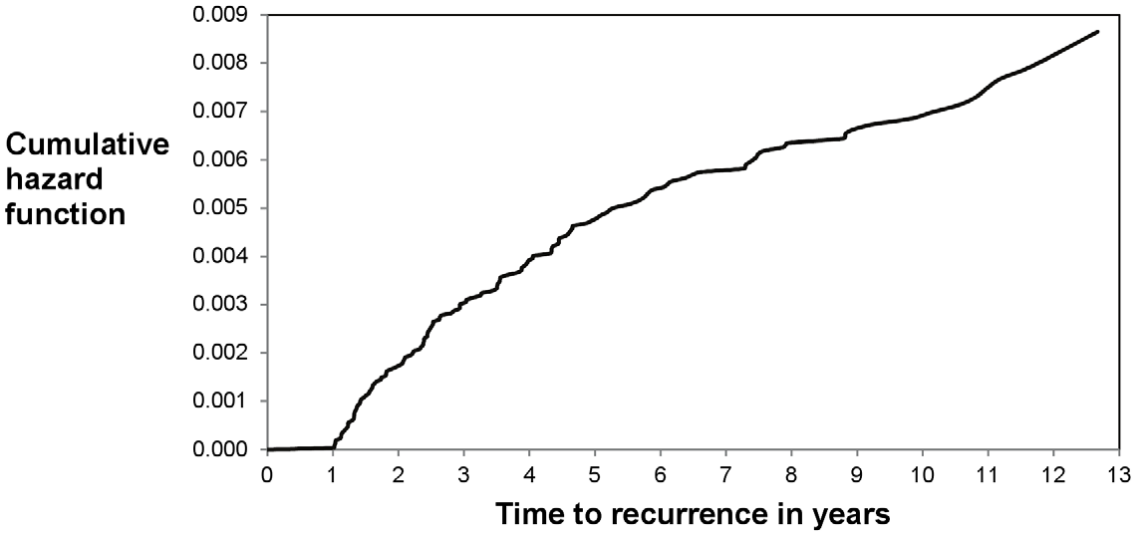
Cumulative hazards of recurrent TB. The figure shows the cumulative hazards of recurrent TB in the study population during the study timeframe. Data for the first one year after treatment completion were not available (see text for details). Of 144 TB patients with complete covariate data, 43 (30%) had a recurrence one to two years, 73 (51%) had a recurrence one to three years, and 108 (75%) had a recurrence one to five years, after completing anti-TB therapy. TB = tuberculosis.

**Figure 4:**
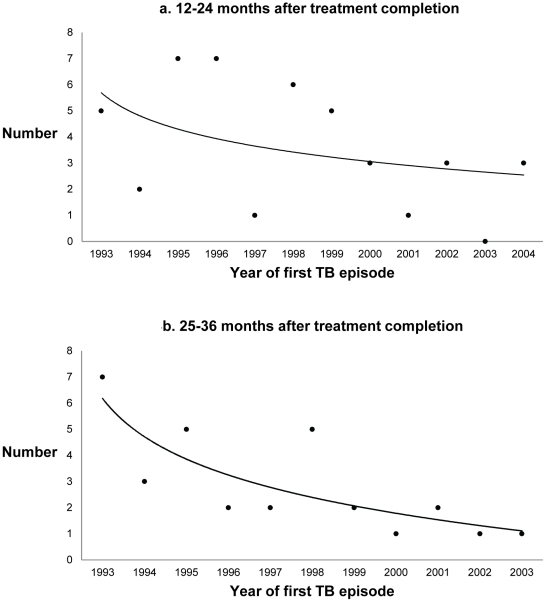
Trends in TB recurrences. Joinpoint regression program-generated trends using Poisson distribution. *a*. Number of persons with a recurrent TB episode 12 to 24 months after treatment completion, by calendar year of first TB episode. The number of TB recurrences appeared to decrease through time, 1993 to 2004, but the trend was not statistically significant. *b*. Number of persons with a recurrent TB episode 25 to 36 months after treatment completion, by calendar year of first TB episode. The number of TB recurrences declined through time, 1993 to 2003, with an annual percent decrease of 15% (p-value<0.05).

Among 23,517 culture-confirmed, pulmonary TB patients who completed anti-TB therapy and were reported in California during 1993–2007, 148 (0.63%) had a second episode of TB reported at least one year after completion of therapy (Figure 2). The majority of late recurrences occurred within three years from treatment completion, and the hazard of recurrence declined with time from treatment completion (Figure 3). Forty-three TB patients had recurrences between months 12 and 24, 30 patients had recurrences between months 25 and 36, and 19 patients had recurrences between months 37 and 48 after completing anti-TB therapy. The number of persons who developed recurrent TB decreased over the study period: patients whose TB recurrence occurred 25 to 36 months after completion of therapy decreased by 15% per year from 1993–2003 (p<0.05, Figure 4b). In addition, the number of patients whose TB recurrence occurred 12 to 24 months after completion of therapy appeared to decline, but the trend was not statistically significant (Figure 4a).

On univariate analyses, several factors were associated with recurrent TB (Tables 1, 2, 3). Compared to persons with only one episode of TB, persons with a late TB recurrence were more likely to have the following characteristics during their initial TB episode: sputum smear-positive disease; cavities on diagnostic chest radiograph; infection with a pyrazinamide mono-resistant isolate of *M. tuberculosis* complex; and HIV co-infection (Table 2). Although persons with late recurrent TB were more likely to have had a documented culture conversion in their first TB episode, compared to persons with one episode of TB (88% vs.79%, p = 0.040), the time to culture conversion was longer for those with late recurrent TB than those with only one TB episode (median = 71 days compared to 54 days, Wilcoxon rank sums test p-value <0.0001).

**Table 1:** Demographic characteristics of patients with late recurrent TB*, California 1993–2007.

	Characteristic	Recurrent TB*n (%)	Single episode TBn (%)	Unadjusted hazard ratio (95% CI)	p-value
No.		148	23,369		
Sex	Male	102 (71)	14,852 (64)	1.41 (0.98–2.02)	0.064
	Female	41 (29)	8,516 (36)	Ref	
Race/Ethnicity	White	16 (11)	2,769 (12)	Ref	
	Black	39 (26)	2,359 (10)	2.86 (1.99–4.13)	<0.0001
	Hispanic	61 (41)	8,251 (35)	1.32 (0.95–1.83)	0.099
	Asian/Pacific Islander	32 (22)	9,835 (42)	0.40 (0.27–0.60)	<0.0001
Age at report, years (median)		42.5	45.0	0.99 (0.99–1.00)	0.145
Birth in U.S.	Yes	65 (44)	6,096 (27)	1.99 (1.44–2.75)	<0.0001
	No	83 (56)	16,878 (73)	Ref	
Homelessness	Yes	26 (18)	1,743 (7)	2.52 (1.65–3.86)	<0.0001
	No	117 (79)	20,876 (89)	Ref	
	Unk	5 (3)	750 (3)		
Substance use†	Yes	43 (29)	3,857 (17)	2.05 (1.42–2.95)	0.0001
	No	87 (59)	17,422 (75)	Ref	
	Unk	18 (12)	2,090 (9)		
Diagnosed in correctional facility	Yes	2 (1)	777 (3)	0.38 (0.09–1.51)	0.168
	No	146 (99)	22,550 (97)	Ref	

Definition of abbreviations: Ref = reference group; Unk = unknown or missing.

*Characteristic present in the first episode of patients with late recurrent TB (see text for details).

† Includes excess alcohol or injection drug use or non-injection drug use.

**Table 2:** Clinical characteristics of patients with late recurrent TB*, California 1993–2007.

	Characteristic	Recurrent TB*n (%)	Single episode TBn (%)	Unadjusted hazard ratio (95% CI)	p-value
No.		148	23,369		
Sputum AFB smear positive	Yes	103 (70)	12,541 (54)	1.97 (1.37–2.83)	0.0002
	No	41 (28)	9,533 (41)	Ref	
	Unk	4 (3)	1,295 (6)		
Cavitary chest radiograph	Yes	51 (34)	5,739 (25)	1.63 (1.16–2.29)	0.0051
	No	94 (64)	16,983 (73)	Ref	
	Unk	3 (2)	647 (3)		
HIV positive	Yes	21 (14)	1,390 (6)	2.46 (1.55–3.90)	0.0001
	No	127 (86)	21,979 (94)	Ref	
INH mono-resistance	Yes	3 (2)	2,040 (9)	0.22 (0.07–0.67)	0.008
	No	145 (98)	21,329 (91)	Ref	
PZA mono-resistance	Yes	5 (3)	354 (2)	2.76 (1.13–6.74)	0.0255
	No	143 (97)	23,015 (98)	Ref	
MDR	Yes	4 (3)	285 (1)	2.56 (0.95–6.92)	0.064
	No	144 (97)	23,084 (99)	Ref	

Definition of abbreviations: Ref = reference group; Unk = unknown or missing; HIV = Human immunodeficiency virus; INH = isoniazid; PZA = pyrazinamide; AFB = acid fast bacilli; MDR = multidrug-resistant.

*Characteristic present in the first episode of patients with late recurrent TB (see text for details).

**Table 3:** Treatment characteristics of patients with late recurrent TB*, California 1993–2007.

	Characteristic	Recurrent TB*n (%)	Single episode TBn (%)	Unadjusted hazard ratio (95% CI)	p-value
No.		148	23,369		
Initial drug regimen	Standard 4drug	120 (81)	19,862 (85)	Ref	0.021
	Two-drug IR	7 (5)	361 (2)	2.46 (1.15–5.26)	0.591
	Other regimen	21 (14)	3,146 (13)	0.88 (0.55–1.40)	
Provider type‡	Health dept	90 (61)	12,552 (54)	1.38 (0.99–1.92)	0.0553
	Private/other	24 (16)	6,445 (28)	Ref	
	Both	34 (23)	4,357 (19)	1.30 (0.88–1.90)	0.186
Anti-TB therapy administration‡	SAT only	51 (34)	7,324 (31)	0.85 (0.60–1.19)	0.343
	DOT only	59 (40)	11,049 (47)	Ref	
	DOT+SAT	35 (24)	4,597 (20)	1.33 (0.91–1.95)	0.137
	Unk	3 (2)	399 (2)		
Time on anti-TB therapy, months (median)		9.02 mo.	8.34 mo.	1.03 (1.0–1.07)	0.052
Documented sputum culture conversion	Yes	130 (88)	18,463 (79)	1.86 (1.03–3.36)	0.0395
	No	12 (8)	2,657 (11)	Ref	
	Unk	6 (4)	2,249 (10)		
Culture conversion within 60 days	Yes	47 (32)	9,306 (40)	Ref	
	No	79 (53)	9,098 (39)	1.58 (1.10–2.26)	0.0137
	Unk	22 (15)	4,965 (21)		

Definition of abbreviations: Ref = reference group; Unk = unknown or missing; Standard 4drug = initial treatment with standard four drug regimen (isoniazid, rifampin, pyrazinamide, ethambutol); Two-drug IR = initial treatment with isoniazid and rifampin only; DOT = directly observed therapy; SAT = self-administered therapy.

*Characteristic present in the first episode of patients with late recurrent TB (see text for details).

‡ 3-level variable comparison.

Of the 148 pairs of *M. tuberculosis* isolates from recurrent TB patients, 147 had isoniazid and rifampin susceptibility test results recorded for first and second TB episodes. One hundred thirty three pairs of isolates (90%) had identical isoniazid and rifampin susceptibility test results for first and second TB episodes: four were MDR; two were isoniazid mono-resistant; and 127 were sensitive to isoniazid and rifampin. Fourteen pairs of isolates (10%) demonstrated changes in initial drug susceptibility patterns from first to second TB episodes; 13 demonstrated increasing resistance, indicating possible re-infection or acquired resistance (Table 4). Genotype data were only available for two pairs of isolates. One of these pairs revealed different DNA fingerprints for each isolate, and one had the same DNA fingerprint.

**Table 4:** Characteristics of patients with late recurrent TB with changes in drug susceptibility patterns from first to second episodes.

Patient	Time between episodes (years)	Initial drug resistance in first episode	Initial drug resistance in second episode
1	1.4	None	INH
2	1.7	None	INH
3	2.9	None	INH
4	5.7	None	INH
5	3.8	None	INH
6	1.2	None	INH
7	5.3	None	RIF
8	1.5	None	RIF
9	5.1	None	RIF
10	2.1	None	RIF
11	1.1	RIF	INH, RIF
12	1.2	None	INH, RIF
13	1.5	None	INH, RIF
14	11.0	INH	None

Definition of abbreviations: INH = isoniazid; RIF = rifampin.

### Risk factors for TB recurrence

Although twenty-one variables had hazard ratios with p-values≤0.20 on univariate analysis (Tables 1, 2, 3), only thirteen variables were tested in the full multivariate Cox proportional hazards model. Five variables were excluded from the full model because they were correlated with other variables. Since three covariates, substance use, non-Hispanic black race, and Asian race, were correlated with birth in the U.S. (phi coefficient = 0.33, 0.47, −0.45 respectively), only birth in the U.S. was included in the multivariate model. Cavities on diagnostic chest radiograph and sputum smear-positive disease are known risk factors for relapse [3] and were correlated (phi coefficient = 0.32). Sputum smear-positive disease had a higher univariate hazard ratio than cavities on diagnostic chest radiograph, and was therefore included in the multivariate model. Cavities on diagnostic chest radiograph was also tested in the full model as a substitute for sputum smear-positive disease and is discussed later. Private provider type was correlated with self-administered therapy (phi coefficient = 0.4). Self-administered therapy was included in the multivariate model because it is a practice that is amenable to public health intervention. Documented culture conversion and culture conversion within 60 days were excluded from the multivariate model due to a high proportion of missing data (Table 3).

The final model identified the following independent risk factors, present in the first TB episode, for late recurrent TB: pyrazinamide mono-resistance (adjusted hazards ratio (aHR) = 2.93, 95% confidence interval (95% CI) = 1.19–7.19, p-value (p) = 0.0190); an initial treatment regimen of isoniazid and rifampin only (aHR = 2.55, 95% CI = 1.04–6.28, p = 0.0412); sputum smear-positive disease (aHR = 1.96, 95% CI = 1.36–2.82, p = 0.0003); birth in the U.S. (aHR = 1.88, 95% CI = 1.34–2.63, p = 0.0002); and HIV co-infection (aHR = 1.81, 95% CI = 1.12–2.91, p = 0.0149). An independent protective factor in the multivariate model was infection with an isoniazid mono-resistant *M. tuberculosis* strain (aHR = 0.25, 95% CI = 0.08–0.78, p = 0.0171). Substituting cavities on diagnostic chest radiograph for sputum smear-positive disease did not substantially change the model (aHR for cavities on chest radiograph = 1.76, 95% CI = 1.24–2.49, p = 0.0015).

The median time-on-treatment was significantly longer for the initial episode of persons with late recurrences than persons with one episode of TB (Table 3). Also, persons with isoniazid or pyrazinamide mono-resistant TB were on treatment for longer periods of time than those with pansusceptible TB (median = 9.4 and 9.9 vs. 7.8 months, respectively, Wilcoxon rank sum test p-values<0.0001 for each comparison). In fact, all persons with late recurrent TB and a pyrazinamide mono-resistant isolate in their first episode were treated for at least nine months. Time-on-treatment was not a statistically significant predictor of late recurrence in the final multivariate model.

We modeled the risk of late TB recurrence, and its timing after treatment completion, among a subpopulation of 30-year-old TB patients with a progressive increase in numbers of risk factors (Table 5). The probability of late TB recurrence one to three years after completing treatment for the first TB episode was 1.42% (95% CI = 0.72–2.1) for 30-year-old U.S.-born, HIV-positive individuals who had sputum smear-positive TB (Table 5). The probability of late TB recurrence one to three years after completing treatment increased to 4.1% (95% CI = 0.1–7.91) for 30-year-old U.S.-born, HIV-positive persons who had sputum smear-positive TB with a pyrazinamide mono-resistant isolate (Table 5).

**Table 5:** Probability of late recurrent TB in a 30-year-old, determined by characteristics of first TB episode: a. after 1 to 13 years, b. after 1 to 3 years post-treatment completion.

Sputum smear status	Nativity	PZA monoR	HIV-negative% (95% CI)	HIV-positive% (95% CI)
Negative	Not U.S.	No	a. 0.61% (0.36–0.85)b. 0.21% (0.13–0.30)	a. 1.09% (0.46–1.72)b. 0.39% (0.16–0.61)
Positive	Not U.S.	No	a. 1.18% (0.80–1.57)b. 0.42% (0.29–0.55)	a. 2.13% (1.00–3.24)b. 0.76% (0.36–1.15)
Positive	U.S.	No	a. 2.21% (1.42–2.99)b. 0.79% (0.50–1.07)	a. 3.96% (2.03–5.85)b. 1.42% (0.72–2.10)
Positive	U.S.	Yes	a. 6.33% (0.27–12.03)b. 2.28% (0.11–4.40)	a. 11.16% (0.22–20.90)b. 4.09% (0.10–7.91)

Definition of abbreviations: PZA monoR = pyrazinamide mono-resistance, CI = confidence interval.

## Discussion

This study is the first large population-based assessment of recurrent TB in the U.S. Of 23,517 pulmonary, culture-positive TB cases in California from 1993–2007, at least 148 (0.63%) recurred after one or more years following treatment completion. Populations at high risk for late recurrence represent a TB control opportunity. Extending patient treatment may prevent recurrent disease and detecting a second TB episode promptly may avert transmission. Risk factors for late TB recurrence, present in the first TB episode, included: infection with a pyrazinamide mono-resistant isolate, initial treatment with isoniazid and rifampin only, sputum smear-positive disease, HIV co-infection, infection with an isoniazid sensitive, rather than an isoniazid mono-resistant isolate, and birth in the U.S.

The proportion of late recurrent TB in California was similar to findings from other surveillance studies and clinical trials in which 0.24% to 0.71% of TB patients had recurrences after twelve months of completing treatment for the first TB episode [6], [9]. However, this comparison may not be relevant because the TB patient follow-up time, and the definition of a TB recurrence in the comparison studies were different. While we cannot differentiate acquired drug resistance from re-infection without additional genotype data, our finding that 13 persons had increased drug resistance in their second TB episode suggests that, at most, only a small proportion (13 episodes = 9% of all recurrences and 0.05% of all study cases) may have been related to drug resistance acquired during treatment. The decline in the frequency of persons with late recurrent TB, as shown by the decreasing number of persons whose TB recurred within one or two years from treatment completion through calendar time, is consistent with declining TB incidence during the study period, and is temporally associated with increased funding for TB programs by Centers for Disease Control and Prevention [10], publication of new guidance for TB control programs [11], and the widespread use of highly active antiretroviral therapy for persons living with HIV [12].

The importance of using recommended TB treatment regimens is confirmed in this study; TB patients who were initiated on only isoniazid and rifampin instead of the recommended four-drug treatment regimen had a 2.5-fold increased risk of recurrence. The use of the recommended four-drug treatment regimen increased during the study timeframe; the proportion of TB cases initiated on the recommended four-drug regimen was 62% in 1993 and 95% in 2006. Since 2000, less than 1% of the study population was initiated on a two drug-only treatment regimen. The increased use of the recommended initial regimen may have also contributed to the decline in late recurrent TB during the study timeframe.

The independent risk factor with the highest hazard ratio was pyrazinamide mono-resistance, a surrogate marker of tuberculosis due to *M. bovis* [13]. Compared to *M. tuberculosis*, *M. bovis* is associated with increased mortality [13], [14], [15], but the current study is the first demonstration of its association with increased TB recurrence. Although resistance to pyrazinamide necessitates treatment extension [2], [16], 26% of patients with pyrazinamide mono-resistant isolates in this study were treated for less than nine months. However, all five patients with pyrazinamide mono-resistant isolates who had late recurrent TB were treated for at least nine months in their first TB episode. Further investigation of treatment strategies to prevent adverse outcomes associated with TB caused by *M. bovis*, including relapse and death, may be warranted.

Our study demonstrated that factors associated with relapse soon after treatment completion were also associated with late recurrence: sputum smear-positive disease, cavities on chest radiograph, and HIV infection [5], [17], [18]. One possible strategy to decrease recurrence is to extend therapy. The combination of cavities on chest radiograph and delayed sputum culture conversion is the primary indication for extended therapy described in current U.S. TB treatment guidelines [2]. Recent evidence has raised questions about the adequacy of six months of rifamycin-based therapy for HIV co-infected patients and suggests further investigation of extended treatment regimens for this group [19], [20].

Our study identified U.S. birth as a risk factor for TB recurrence. Birth in the U.S. may have been a proxy for increased risk of non-adherence to anti-TB therapy [21], increased risk for re-exposure (e.g. among homeless or substance abusers), or other unmeasured risk factors for recurrence (e.g. smoking) [22], [23], [24], [25]. An unexpected finding of this study was the protective effect of isoniazid mono-resistant TB. It is possible that isoniazid mono-resistant TB was a proxy for the use of six months of anti-TB therapy with pyrazinamide, recommended by national guidelines [2], or, of extension of standard anti-TB drug therapy [26].

Our findings should be interpreted in light of a number of study limitations. We had no information about the true length of follow-up (person-time at risk) for individuals who had a single TB episode. The use of the censor date provided an approximation that may have overestimated length of follow-up for the TB patient population, resulting in an increasing underestimate of the hazard of late recurrent TB over time. Also, the absolute number of persons who developed a late recurrence is an underestimate because some unmeasured number of persons may have developed a second episode of TB after moving out of California. The absence of sufficient genotyping data to assess the relative frequency of relapse and re-infection in this population limits inferences about acquired drug resistance and the evidence base for recommending specific prevention interventions. Because this study used the time between treatment initiation and treatment completion dates as a surrogate for the time-on-treatment covariate, the extent and duration of treatment interruptions were unknown and likely differed by risk groups. Indeed, persons with sputum smear-positive disease, HIV infection, or infection with pyrazinamide mono-resistant isolates of *M. tuberculosis* were more likely to have a longer time-on-treatment than persons without these characteristics. Thus, the absence of evidence for time-on-treatment as a predictor of late recurrent TB in this population may reflect the limitation of the available data rather than the actual lack of influence of treatment duration in the first TB episode. Finally, the HIV status determination may have been incomplete, leading to an underestimate of HIV co-infection frequency in this population. However, we expect no difference in the extent of TB/HIV co-infection underestimation for first episodes of individuals with recurrent TB compared to individuals with one episode of TB.

How should healthcare providers address the elevated risk of TB recurrence in specific subpopulations? Post-treatment monitoring beyond one year may facilitate early TB case-finding among those at high risk of recurrence, but clinical trial evidence to support this intervention is limited. Implementation of post-treatment monitoring varies in California, by local TB programs, and elsewhere in the U.S. In one public health jurisdiction, TB clinic appointments at six- but not twelve- months after treatment completion facilitated early case-finding [27], suggesting that the effectiveness of post-treatment monitoring may wane as the time from treatment completion increases.

On the other hand, there is precedent for routine post-treatment follow-up of TB patients at increased risk of recurrence. Individuals who complete treatment for MDR-TB are monitored for at least 24 months to identify potential relapse [16]. Also, U.S. guidelines recommend close post-treatment monitoring of HIV-infected patients who are at high risk of re-exposure to TB [28]. Additional data are needed to assess the effectiveness of different post-treatment follow-up strategies to detect recurrent TB early and prevent transmission.

In summary, this study provides a minimal estimate of the frequency of late TB recurrence in a low incidence setting, and suggests populations that may benefit from additional medical and public health interventions or further investigation. Additionally, our findings raise questions about the adequacy of treatment for HIV-positive TB patients and TB patients with pyrazinamide mono-resistant isolates. Investigation of treatment extension and the utility of extended follow-up in specific patient groups at high risk of recurrence may be warranted.
